# A first in human, safety, pharmacokinetics, and clinical activity phase I study of once weekly administration of the Hsp90 inhibitor ganetespib (STA-9090) in patients with solid malignancies

**DOI:** 10.1186/1471-2407-13-152

**Published:** 2013-03-25

**Authors:** Jonathan W Goldman, Robert N Raju, Gregory A Gordon, Iman El-Hariry, Florentina Teofilivici, Vojo M Vukovic, Robert Bradley, Michael D Karol, Yu Chen, Wei Guo, Takayo Inoue, Lee S Rosen

**Affiliations:** 1 UCLA Medical Center, Suite 600, 2020 Santa Monica Blvd, Santa Monica, CA 90404, USA; 2 Kettering Health Network Innovation Center, 3535 Southern Blvd, Kettering, OH 45429, USA; 3 Synta Pharmaceuticals Corp., 125 Hartwell Ave, Lexington, MA 02421, USA

**Keywords:** Ganetespib, Hsp90 inhibitor, Pharmacokinetics, Phase I study, Solid tumors

## Abstract

**Background:**

This phase I study investigated the maximum tolerated dose (MTD), safety, pharmacokinetics and antitumor activity of ganetespib in patients with solid malignancies.

**Methods:**

Patients were enrolled in cohorts of escalating ganetespib doses, given as 1 hour IV infusion, once weekly for 3 weeks, followed by a 1-week rest until disease progression or unacceptable toxicity. Endpoints included safety, pharmacokinetic and pharmacodynamic parameters and preliminary clinical activity.

**Results:**

Fifty-three patients were treated at doses escalating from 7 to 259 mg/m^2^. The most common adverse events were Grade 1 and 2 diarrhea, fatigue, nausea or vomiting. Dose-limiting toxicities (DLT) observed were: one Grade 3 amylase elevation (150 mg/m^2^), one Grade 3 diarrhea and one Grade 3 and one Grade 4 asthenia (259 mg/m^2^). The MTD was 216 mg/m^2^ and the recommended phase 2 dose was established at 200 mg/m^2^ given IV at Days 1, 8, and 15 every 4 weeks. There was a linear relationship between dose and exposure. Plasma HSP70 protein levels remained elevated for over a week post treatment. Disease control rate (objective response and stable disease at ≥ 16 weeks) was 24.4%.

**Conclusions:**

Ganetespib is well tolerated as a weekly infusion for 3 of every 4 weeks cycle. The recommended phase II dose is 200 mg/m^2^, and is associated with an acceptable tolerability profile.

**Trial registration:**

NCT00687934

## Background

Heat shock protein 90 (Hsp90) belongs to a class of molecular chaperone proteins that helps modulate cellular responses to environmental stress, and regulates the folding, stability, and function of many so-called “client” proteins, such as *RAF*, *KIT*, *EGFR*, *HER2*, *PDGFRα* and *VEGFR2*[1]. These client proteins play critical roles in tumor growth, evasion of apoptosis, angiogenesis, and tissue invasion [2-4]. Inhibition of Hsp90 is believed to cause these client proteins to adopt aberrant conformations, which are then targeted for ubiquitination and degradation by the proteasome, thereby providing simultaneous targeting of multiple oncogenic signaling pathways [5-7]. In addition to client protein degradation, induction of heat shock protein 70 (HSP70) is another feature of Hsp90 inhibition. HSP70 is also a molecular chaperone that is known to play a key role in the Hsp90 chaperone complex machinery [8,9]. In this regard, HSP70 up-regulation is a commonly used biomarker for Hsp90 inhibition in clinical trials [10]. In most cases, pharmacodynamic analyses of Hsp90 inhibitors have focused on cytosolic HSP70 levels using circulating peripheral blood mononuclear cells (PBMCs) as a surrogate tissue for tumor-specific activity. However, because HSP70 has been documented to be secreted by tumor cells and elevated in the sera of cancer patients, plasma levels of HSP70 have been proposed to represent a potentially more robust and reproducible biomarker for Hsp90 inhibition [11].

**Figure 1:**
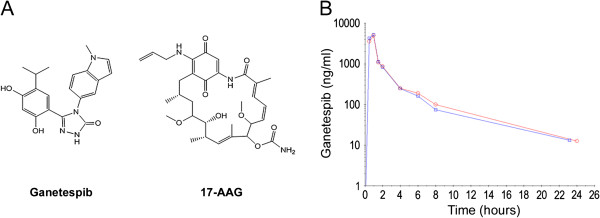
**Chemical structures of Hsp90 inhibitors and ganetespib concentration vs. time curves.** (**A**) Ganetespib (left) and 17-AAG, a prototypical geldanamycin-derived Hsp90 inhibitor (right); (**B**) Representative ganetespib concentration vs. time curves for a 216 mg/m2 dose. Red circles represent Day 1, blue squares represent Day 15.

Ganetespib (STA-9090), 5-[2,4-dihydroxy-5-(1 methyl-ethyl)phenyl]-2,4 dihydro-4-(1-methyl-1*H* indol-5 yl)-3*H*-1,2,4 triazole-3-one, is a novel triazolone heterocyclic Hsp90 inhibitor [12], structurally unrelated to geldana-mycin-derived inhibitors such as 17-AAG, 17-DMAG and IPI-504 (Figure 1A). Ganetespib exerts its action by binding to the ATP pocket in the N-terminus of Hsp90, leading to down-regulation of Hsp90 client protein levels. Preclinical studies reveal potent Hsp90 inhibition and activity against a range of models including lung, prostate, colon, breast, melanoma and leukemia [13-15]. In non-small cell lung cancer (NSCLC) models in particular, ganetespib effectively destabilizes a number of oncogenic drivers, including the KRAS effector CRAF and PDGFRα, that in turn inactivates downstream MAPK and AKT signaling to induce apoptosis [16]. In combination with taxanes, ganetespib is also highly efficacious in NSCLC models that express the activated and erlotinib resistant form of the epidermal growth factor receptor (EGFR^L858R/T790M^) [17].

This study was undertaken to determine the maximum tolerated dose (MTD), and the recommended phase II dose (RP2D) in solid tumors.

## Methods

### Study design

This open-label, dose-escalation study was conducted at 2 centers (Premiere Medical Center [currently UCLA, Santa Monica, CA] and KHN Innovation Center, US Oncology, Kettering, OH). The primary objectives were to charac-terize the safety and tolerability of a once-weekly administration, determine the recommended phase II dose (RP2D) of ganetespib, pharmacokinetics (PK), pharmacodynamics (PD), and preliminary clinical activity. The study was approved by the Institutional Review Board at both centers and was carried out in accordance with Good Clinical Practice.

### Eligibility criteria

Eligible patients had pathologically confirmed advanced solid tumors, whose disease was refractory to prior therapies or for whom no further standard therapy existed. Patients were required to be ≥ 18 years of age; with Eastern Cooperative Oncology Group (ECOG) performance status (PS) ≤ 2; adequate hematologic, renal and hepatic functions; and left ventricular ejection fraction (LVEF) greater than 45%. Measurable disease was not required for entry. Primary brain tumors were excluded, but patients with stable brain metastases were eligible. All patients gave written informed consent according to institutional and federal guidelines.

### Study assessments

Patients’ demographics and medical history were recorded at baseline. Physical examination and PS were assessed at baseline and on Day 1 of each cycle. Adverse events (AEs), vital signs, hematology and chemistry values, and creatinine clearance were assessed at baseline and weekly during treatment. Toxicity was graded using National Cancer Institute Common Terminology Criteria for Adverse Events (NCI CTCAE), version 3.0. An electrocardiogram (ECG) was performed at baseline, before and after treatment on Days 1 and 15 of Cycles 1 and 2, and on Day 15 of even-numbered cycles thereafter. CT scans were done at baseline and every 8 weeks thereafter. Tumor response was assessed using Response Evaluation Criteria in Solid Tumors (RECIST, v1.0), with confirmation of responses performed at least 4 weeks later.

### Treatment and dose escalation

Ganetespib was administered over a 1-hour infusion, once weekly for 3 weeks of a 4-week cycle. Intra-patient dose escalation was allowed to dose levels shown to be safe and tolerable. The starting dose (7 mg/m^2^) was selected based on a conservative estimate using the highest non-severely toxic dose established in a once-weekly, 4-week repeat dose study in cynomolgus monkeys. Dose escalation followed a modified Fibonacci design resulting in levels that escalated from 7 mg/m^2^ to 14, 23, 35, 49, 65, 86, 114, 150, 180, 216, and 259 mg/m^2^. Each cohort consisted of 3 patients, with expansion to 6 patients if 1 of the 3 initial patients experienced a DLT, which was defined as: Grade 4 thrombocytopenia (or Grade 3 with hemorrhage); Grade 4 neutropenia lasting > 7 days (or Grade 3 with fever); Grade 4 anemia; ≥ Grade 3 non-hematologic toxicity (except alopecia); and ≥ Grade 3 hypersensitivity despite premedication. Doses were escalated after all patients in the preceding dose cohort had completed Cycle 1. Dose reductions and delays of up to 14 days were allowed for recovery from toxicity. The RP2D was defined as the dose of ganetespib below which 2 of 3 or 2 of 6 patients experienced a DLT. Once the RP2D was determined, the respective cohort was expanded up to 12 patients, to further define the safety and pharmacokinetic profile.

### Pharmacokinetic and pharmacodynamic analyses

Blood samples were taken for ganetespib plasma concentration determination on Days 1 and 15 of Cycle 1 pre-dose, 0.5, 1, 1.5, 2, 4, 6, 8 and 24 h after infusion initiation. Samples were also drawn pre-dose and at 1 h, on Day 8 of Cycle 1 and Days 1, 8 and 15 of subsequent cycles. Plasma was separated and stored at a −70°C until analysis. Analyses were performed by a validated HPLC-MS/MS method under GLP conditions at Synta Pharmaceuticals Corp. Calibration curve coefficients of determination (r^2^) ranged from 0.9897 to 0.9992. Back-calibrated calibration standards were in good agreement with QC samples with bias ≤ ± 3%, and calibration-curve r^2^ variation was ≤ ± 6.5% across a concentration range of 0.100 through 100 ng/ml.

Pharmacokinetic parameters were computed non-compartmentally using standard methods within a validated installation of WinNonlin (v4.1, Pharsight Corporation, St. Louis, MO). Parameters included the maximum concentration (C_max_), area under the plasma concentration versus time curve (AUC), time of maximum concentration (T_max_), and terminal elimination half-life (t_1/2_).

Pre-dose blood samples on Days 1, 8 and 15 of Cycle 1 and 2 were collected for assessment of HSP70 protein in plasma by ELISA. Assays were performed using high sensitivity HSP70 ELISA kits (Assay Design, Ann Arbor, Michigan), with a sensitivity limit as low as 90 pg/ml, according to manufacturers’ instructions. Results were detected using a microplate ELISA reader at 450 nm with a correction wavelength of 540 nm. Concentrations of HSP70 were normalized to the total protein in each plasma sample.

No tumor biopsies were requested as part of the study however archival tumor samples, collected prior to ganetespib treatment, were available from a limited number of patients. From those individuals with available tissue, gene mutational analysis was carried out on DNA extracted from archived tumor samples on the Sequenom MassARRAY platform (53 genes; 649 mutations) according to the manufacturer’s protocol.

## Results

### Patient characteristics

Fifty-three patients were enrolled in the study between January 2008 and January 2010 and treated at doses escalating from 7 to 259 mg/m^2^. For purposes of data analyses, dose levels were grouped to three cohorts: 7–114 mg/m^2^ (*n* = 25), 150–216 mg/m^2^ (*n* = 22), and 259 mg/m^2^ (*n* = 6); and their baseline characteristics are shown in Table 1. All 53 patients were included in the analyses. However there were 6 patients who retrospectively did not meet the eligibility criteria, due to abnormal baseline hematological and serum chemistry (*n* = 2, one patient enrolled at 7 mg/m^2^ and one enrolled at 216 mg/m^2^ dose levels), insufficient cardiac function (*n* = 1, enrolled at 216 mg/m^2^ dose level), or incomplete recovery from prior therapies (*n* = 3, two patients enrolled at 35 mg/m^2^ dose level, and one at 216 mg/m^2^ dose level). The study population included patients with a variety of solid tumors, with NSCLC being the most common (*n* = 10). The majority of patients were heavily pre-treated, with 32 patients (60.3%) receiving at least 3 prior systemic therapies.

**Table 1:** Patients’ characteristics at baseline

	**Number (%) of patients**			
	**Ganetespib**	**Ganetespib**	**Ganetespib**	**All**
	**7-114 mg/m**^**2**^	**150-216 mg/m**^**2**^	**259 mg/m**^**2**^	**patients**
	***n*** **= 25 (%)**	***n*** **= 22 (%)**	***n*** **= 6 (%)**	***n*** **= 53 (%)**
Age (years)				
Median (range)	61 (39, 87)	62 (37, 80)	61 (47, 81)	61 (37, 87)
Sex, *n* (%)				
Female	14 (56)	7 (31.8)	3 (50)	24 (45.3)
Male	11 (44)	15 (68.2)	3 (50)	29 (54.7)
Race, *n* (%)				
White	24 (96)	19 (86.4)	6 (100)	49 (92.5)
Black	0	2 (9.1)	0	2 (3.8)
Other	1 (4)	1 (4.5)	0	2 (3.8)
ECOG PS, *n* (%)				
0	10 (40)	5 (22.7)	2 (33.3)	17 (32.1)
1	13 (52)	17 (77.3)	4 (66.7)	34 (64.2)
2	2 (8)	0	0	2 (3.8)
Primary tumor site, *n* (%)				
NSCLC	5 (20)	3 (13.6)	2 (33.3)	10 (18.9)
colorectal	2 (8)	6 (27.3)	0	8 (15.1)
Prostate	3 (12)	0	0	3 (5.7)
Esophageal	1 (4)	2 (9.1)	0	3 (5.7)
SCLC	1 (4)	1 (4.5)	0	2 (3.8)
Pancreas	1 (4)	1 (4.5)	0	2 (3.8)
Ovarian	2 (8)	0	0	2 (3.8)
Others	10 (40)	9 (40.9)	4 (66.7)	23 (43.3)
Tumor stage at study entry, *n* (%)				
III	2 (8)	0	0	2 (3.8)
IV	23 (92)	22 (100)	6 (100)	51 (96.2)
Number of prior systemic therapies, *n* (%)				
0	1 (4)	1 (4.5)	0	2 (3.8)
1	0	2 (9.1)	1 (16.7)	3 (5.7)
2-3	7 (28)	6 (27.3)	3 (50)	16 (30.2)
≥ 3	17 (68)	13 (59.1)	2 (33.3)	32 (60.4)

ECOG PS, Eastern Cooperative Oncology Group Performance Status.

### Study treatment

All patients in the study received at least one dose of ganetespib, with 5 patients (9.4%) receiving ≥ 8 cycles. Three subjects (5.7%) dose-escalated without complication. Dose modification was observed in 24 patients (45.3%): missed dose (*n* = 16, 30.2%), dose reduction (*n* = 4, 7.5%), or dose reduction and delay (*n* = 4, 7.5%), all mainly due to adverse events. Three patients (5.7%), all in cohort 1, discontinued ganetespib treatment due to drug-unrelated adverse events: one patient with endometrial carcinoma had hepatic failure that led to her death; one patient with small cell lung cancer (SCLC) had spinal cord compression; and one patient with esophageal cancer had biliary obstruction.

### Recommended phase II dose

None of the patients in the 7–114 mg/m^2^ cohort experienced DLT, and therefore dose was escalated to next dose levels. At the 150 mg/m^2^ dose level, one patient experienced a DLT of asymptomatic, transient Grade 3 elevated serum amylase. This dose level was expanded to 6 patients with a 7^th^ being added as one patient was deemed not evaluable for dose escalation. No further DLT was observed at that dose level or the subsequent 180 mg/m^2^ (*n* = 3) and 216 mg/m^2^ (*n* = 6) doses. The 216 mg/m^2^ cohort was expanded to 6 patients due to an Investigator assessment of Grade 3 QTc prolongation. A subsequent independent cardiology review revealed technical factors that were deemed the likely cause of the ECG findings. Possible confounding factors included automated machine-read ECG QT intervals that could not be duplicated upon expert cardiologist’s over read; variation in lead placement; and the use of Bazett’s correction formula, a method prone to over and under correction. Based on this information, the Investigator updated his assessment and without QTc prolongation, the event was not deemed a DLT. At the 259 mg/m^2^ dose level, two patients experienced DLTs of Grade 3 and 4 asthenia, and the dose level was expanded to 6 patients, with one additional patient experiencing DLT of repeated Grade 3 diarrhea.

The 216 mg/m^2^ dose level was subsequently declared the MTD and was further expanded with 6 additional patients. One patient experienced Grade 3 fatigue, which would have been considered dose-limiting in the dose-escalation phase. The criteria for MTD of ≥ 2 out of 6 patients were not met, and therefore did not affect the establishment of the phase II dose. The dose was rounded to 200 mg/m^2^ as the ganetespib RP2D administered on Days 1, 8, 15 of a 28 day cycle.

### Toxicity

All patients experienced at least one AE. The most common toxicities reported during the study treatment are listed in Table 2, and were diarrhea and fatigue, with Grade 1 and 2 reported in 47 (88.7%) and 30 (56.6%) patients, respectively. The incidence of diarrhea and fatigue increased with higher ganetespib doses (7–114 mg/m^2^ dose levels: 80% and 48%; 150–216 mg/m^2^ dose levels: 95.5% and 59.1%; and 259 mg/m^2^ dose level: 100%, and 83.3%, respectively). In most patients (*n* = 40; 75.5%), the onset of diarrhea occurred between days 1–7, and generally resolved with anti-diarrheal treatment. Other frequent AEs were mainly gastrointestinal, such as abdominal pain (*n* = 20; 37.7%), nausea (*n* = 18; 34%) and vomiting (*n* = 10; 18.9%), and were mild to moderate.

**Table 2:** Adverse events of any grade reported in 10% or more patients during study treatment, regardless of causality

	Number (%) of patients*			
	**Ganetespib**	**Ganetespib**	**Ganetespib**	**All**
	**7-114 mg/m**^**2**^	**150-216 mg/m**^**2**^	**259 mg/m**^**2**^	**patients**
	***n*** **= 25 (%)**	***n*** **= 22 (%)**	***n*** **= 6 (%)**	***n*** **= 53 (%)**
Any event	25 (100)	22 (100)	6 (100)	53 (100)
Diarrhea	20 (60)	21 (95.5)	6 (100)	47 (88.7)
Fatigue	12 (48)	13 (59.1)	5 (83.3)	30 (56.6)
Abdominal pain	9 (36)	10 (45.5)	1 (16.7)	20 (37.7)
Nausea	7 (28)	7 (31.8)	4 (66.7)	18 (34)
Anemia	11 (44)	5 (22.7)	0	16 (30.2)
Decreased appetite	2 (8)	8 (36.4)	1 (16.7)	11 (20.8)
ALT elevated	5 (20)	5 (22.7)	0	10 (18.9)
Insomnia	2 (8)	6 (27.3)	2 (33.3)	10 (18.9)
Vomiting	4 (16)	3 (13.6)	3 (50)	10 (18.9)
AST elevated	5 (20)	4 (18.2)	0	9 (17)
Constipation	5 (20)	3 (13.6)	1 (16.7)	9 (17)
Dyspnea	4 (16)	4 (18.2)	1 (16.7)	9 (17)
Headache	2 (8)	6 (27.3)	1 (16.7)	9 (17)
Peripheral edema	3 (12)	5 (22.7)	1 (16.7)	9 (17)
Asthenia	2 (8)	4 (18.2)	2 (33.3)	8 (15.1)
Back pain	4 (16)	2 (9.1)	2 (33.3)	8 (15.1)
Urinary tract infection	2 (8)	3 (13.6)	3 (50)	8 (15.1)
Dehydration	1 (4)	3 (13.6)	3 (50)	7 (13.2)
Hypokalemia	2 (8)	4 (18.2)	1 (16.7)	7 (13.2)
Hypophosphatemia	5 (20)	2 (9.1)	0	7 (13.2)
Weight decreased	1 (4)	5 (22.7)	1 (16.7)	7 (13.2)
Abdominal distension	3 (12)	3 (13.6)	0	6 (11.3)
ALT elevated	3 (12)	3 (13.6)	0	6 (11.3)
Dizziness	1 (4)	3 (13.6)	2 (33.3)	6 (11.3)
Dry mouth	2 (8)	3 (13.6)	1 (16.7)	6 (11.3)
Musculoskeletal chest pain	2 (8)	4 (18.2)	0	6 (11.3)
Extremity pain	4 (16)	1 (4.5)	1 (16.7)	6 (11.3)
Rash	4 (16)	1 (4.5)	1 (16.7)	6 (11.3)

*A patient may have had more than one event.

Elevated hepatic enzymes were infrequent and generally Grade 1 or 2. Ten (18.9%), 9 (17%), and 6 (11.3%) patients had transient ALP, AST, and ALT elevation, respectively. Four patients (7.5%) had Grade 2 or 3 hyberbilirubinemia; however, the events were not considered study drug-related, as most of these patients presented with extensive hepatic metastases.

Eight patients (15%) had visual changes, which were mild and transient. Three patients experienced Grade 1 or 2 blurred vision at doses of 35 mg/m^2^, 114 mg/m^2^ and 150 mg/m^2^. Grade 1 transient visual impairment was reported in 2 patients (one each at doses of 216 and 259 mg/m^2^) each case considered to be possibly related to study drug. Other changes were Grade 1 conjunctivitis, eyelid edema, and night blindness, which were study drug-unrelated.

One patient with a history of coronary artery disease had Grade 1 atrio-ventricular block at 259 mg/m^2^, which was possibly related to study drug. Three patients experienced QTc prolongation at higher dose levels on Cycle 1 Day 1 post-dose when QT = 438 ms, and QTc = 457 (QT with Bazett correction); however, a repeat ECG performed later on the same day showed resolution of the reported changes, with QT = 414 ms and QTc = 433. QTc changes were reported in 48 patients (91%) that were not symptomatic, did not lead to brady-arrhythmias, and were not considered clinically meaningful by an independent cardiologist who reviewed the ECG data. No clinically significant changes were detected in the vital sign measurements at any dose level.

The most common hematological toxicities considered by the investigators to be treatment-related were anemia and neutropenia, occurring in 3 (5.7%) patients each.

A total of 36 patients (67.9%) experienced Grade 3 or 4 AE at some point in their participation, with fatigue being the most commonly reported event (*n* = 6, 11.3%) (Table 3).

**Table 3:** Incidence of CTCAE Grade 3 and 4 adverse events (occurring in ≥ 2 patients), regardless of causality

	Number (%) of patients*			
	**Ganetespib**	**Ganetespib**	**Ganetespib**	**All**
	**7-114 mg/m**^**2**^	**150-216 mg/m**^**2**^	**259 mg/m**^**2**^	**patients**
	***n*** **= 25 (%)**	***n*** **= 22 (%)**	***n*** **= 6 (%)**	***n*** **= 53 (%)**
Any event	17 (68)	14 (63.6)	5 (83.3)	36 (67.9)
Fatigue^†^	1 (4)	4 (18.2)	1 (16.7)	6 (11.3)
Asthenia^†^	1 (4)	1 (4.5)	2 (33.3)	4 (7.5)
Diarrhea ^†^	1 (4)	2 (9.1)	1 (16.7)	4 (7.5)
Hypophosphatemia	2 (8)	2 (9.1)	0	4 (7.5)
ALT elevation^†^	2 (8)	1 (4.5)	0	3 (5.7)
Dehydration	1 (4)	1 (4.5)	1 (16.7)	3 (5.7)
Hyperbilirubinemia	1 (4)	2 (9.1)	0	3 (5.7)
Hyponatremia	3 (12)	0	0	3 (5.7)
Arthralgia	1 (4)	0	1 (16.7)	2 (3.8)
Serum amylase elevated^†^	1 (4)	1 (4.5)	0	2 (3.8)
Hypokalemia^†^	0	2 (9.1)	0	2 (3.8)
Spinal cord compression	2 (8)	0	0	2 (3.8)

*A patient may have had more than one event.

^†^Considered by the investigator to be clinically significant.

The number of patients with on-treatment SAEs is shown in Table 4. None of the observed SAEs (*n* = 15, 28.3%) were considered treatment-related.

**Table 4:** Number (%) of patients with serious adverse events (that affected ≥ 2 patients)

	Number (%) of patients*			
	**Ganetespib**	**Ganetespib**	**Ganetespib**	**All**
	**7-114 mg/m**^**2**^	**150-216 mg/m**^**2**^	**259 mg/m**^**2**^	**patients**
	***n*** **= 25 (%)**	***n*** **= 22 (%)**	***n*** **= 6 (%)**	***n*** **= 53 (%)**
Any event	6 (24)	6 (27.3)	3 (50)	15 (28.3)
Abdominal pain	0	1 (4.5)	1 (16.7)	2 (3.8)
Asthenia	0	1 (4.5)	1 (16.7)	2 (3.8)
Dehydration	0	1 (4.5)	1 (16.7)	2 (3.8)
Pneumonia	0	1 (4.5)	1 (16.7)	2 (3.8)

*A patient may have had more than one event.

Three deaths were reported during the study; none was deemed to be treatment-related. The causes of death were hepatic failure, intestinal obstruction, and respiratory failure.

### Clinical activity

Forty-two patients were evaluable for clinical activity, and 11 patients discontinued treatment before first disease assessment (Table 5). One patient with metastatic colorectal cancer had a PR, and 23 patients (43.4%) had SD (range 46–563 days). No tumor tissue was available from the patient achieving the PR, hence the mutational status of this tumor was unknown. Disease control rate (PR and SD ≥ 16 weeks) was 24.5%.

**Table 5:** Investigator-evaluated assessment of best overall response

	**Ganetespib**	**Ganetespib**	**Ganetespib**	**Overall**
	**7-114 mg/m**^**2**^	**150-216 mg/m**^**2**^	**259 mg/m**^**2**^	**response**
	***n*** **= 25 (%)**	***n*** **= 22 (%)**	***n*** **= 6 (%)**	***n*** **= 53 (%)**
Best response^a^, *n* (%)				
Complete response	0	0	0	0
Partial response	1 (4)	0	0	1 (1.9)
Stable disease	10 (40)	11 (50)	2 (33.3)	23 (43.4)
Progressive disease	10 (40)	5 (22.7)	3 (50)	18 (34)
Non-evaluable^b^	4 (16)	6 (27.3)	1 (16.7)	11 (20.8)
Disease control rate (≥ 8 weeks)^c^	11 (44)	11 (50)	2 (33.3)	24 (45.3)
Disease control rate (≥ 16 weeks)^d^	5 (20)	6 (27.3)	2 (33.3)	13 (24.5)

^a^Initial assessment at 8 weeks from treatment start with confirmation assessment at least 4 weeks later.

^b^Reasons for non-evaluable patients: investigator decision (2), symptom deterioration (8) and withdrawal of informed consent (1).

^c^disease control rate: Complete and partial responses, and stable disease ≥ 8 weeks.

^d^disease control rate: Complete and partial responses, and stable disease ≥ 16 weeks.

A total of 10 patients presented with NSCLC; of these 6 patients (60%) had SD for at least 8 weeks. One patient receiving ganetespib at 150 mg/m^2^ had a maximum reduction in target lesions of 26.5% and remained on study for 13 months. Molecular profiling revealed a BRAF G469A mutation. For this individual, circulating plasma HSP70 levels increased following ganetespib dosing and remained elevated during both treatment cycles, peaking at 750 and 730 ng/g in Cycles 1 and 2, respectively.

Another patient with metastatic GIST receiving ganetespib at 216 mg/m^2^ attained durable disease stabi-ization with a maximum reduction in target lesions of 18%. Mutational analysis showed *PDGFRA*^*D842V*^ exon 18 mutation.

One patient diagnosed with neuroendocrine tumor was treated with ganetespib (259 mg/m^2^) and achieved disease stabilization over 20 months. However, gene mutational analysis was inconclusive.

### Pharmacokinetics

Ganetespib concentration rose rapidly during infusion and declined rapidly upon termination. The concentration of ganetespib declined to approximately 10% of C_max_ within 1 h of infusion termination and 1% of C_max_ within 8 to 10 h (Figure 1B). Day 1 and 15 concentration profiles were similar and there was no apparent drug accumulation for these once-weekly doses.

**Figure 2:**
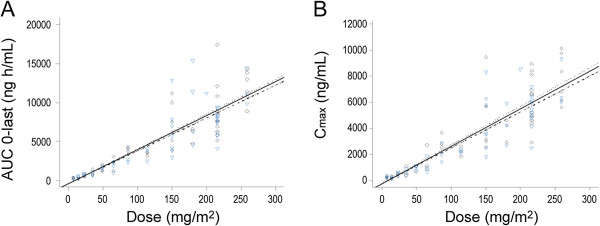
**Pharmacokinetic linearity plots.** (**A**) AUC vs. Dose and (**B**) C_max_ vs. Dose. Diamonds represent Day 1, triangles represent Day 15. Solid line represents linear regression of Day 1 and Day 15 data combined. Dotted line is Day 1 only. Dashed and dotted line is Day 15 only. For Days 1 and 15 combined, coefficients of determination for AUC and C_max_ were 0.7547 and 0.7637, respectively.

The mean ± SD terminal t_1/2_ was approximately 7.54 ± 2.64 h and plasma drug clearance was 52.59 ±17.80 L/h or 28.55 ± 9.33 L/h/m^2^. Mean T_max_ was at 0.79 h. During infusion samples were drawn at 0.5 and 1 h. T_max_ occurrence at the time of the 0.5 h sample in 39% of drug administrations is consistent with a rapid alpha phase and suggests that the drug achieves near maximal concentrations within the first 30 min of infusion initiation (Figure 1B). Mean steady state volume of distribution (V_ss_) was 196 ± 172 L or 107 ± 98 L/m^2^. Clearance and volume of distribution were approximately constant across doses. AUC increased in proportion to dose for each of Days 1 and 15 (Figure 2A). The relationship of AUC to dose for the two days was essentially identical, as shown in the individual-day regression lines. As such, the data from Days 1 and 15 were combined to provide a single descriptor of AUC versus dose. The coefficient of determination (r^2^) was 0.7547.

C_max_ also increased in relative proportion to dose, with Day 1 and 15 being similar (Figure 2B). Linear regression of the combined data from Days 1 and 15 gave an r^2^ value of 0.7367. Indeed, ganetespib C_max_ was an excellent predictor of AUC, with a coefficient of determination of 0.9270. Regression analysis also suggested that there were no statistically significant associations between C_max_ or AUC and diarrhea (*P* = 0.17).

### Pharmacodynamics

**Figure 3:**
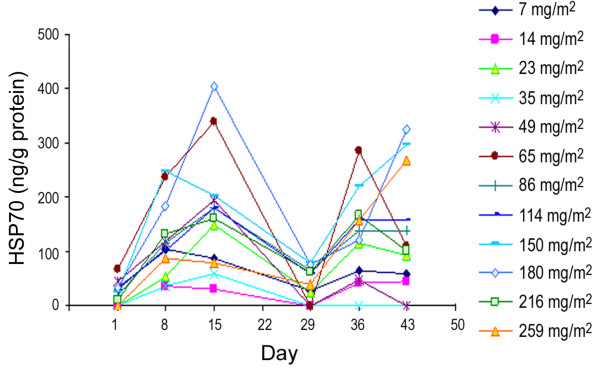
**Plasma HSP70 protein concentrations on days 1, 8 and 15 of Cycle 1 for 7–114 mg/m**^**2 **^**and 150–259 mg/m**^**2 **^**dose groups.**

For a majority of the patients evaluated, baseline Hsp70 plasma protein levels were low, but were significantly elevated on Days 8 and 15 (immediately prior to the second and third administration of ganetespib, respectively). This increase in response to ganetespib administration is indicative of ganetespib bioactivity in patients and, importantly, biological responsiveness to ganetespib was retained during the second treatment cycle. Elevated HSP70 protein plasma levels persisted for at least a week following drug exposure. Additionally, the higher mean maximum increase of HSP70 observed in Cycle 1 suggested that Hsp70 induction saturates at dose levels above 180 mg/m^2^, further supporting the selection of the 200 mg/m^2^ dose for Phase 2 (Figure 3). There was no statistically significant association between HSP70 induction and DCR at 8 weeks (*P* < 0.79), or with diarrhea incidence (*P* < 0.81).

## Discussion

We report here the first-in-human phase I study of ganetespib administered once weekly for 3 weeks of a 4-week cycle. This study demonstrated dose-proportional pharmacokinetics and tolerability at doses ranging from 7 mg/m^2^ to 216 mg/m^2^ in patients with advanced solid malignancies. There were no DLTs in the 216 mg/m^2^ dose escalation cohort, and therefore, this dose was rounded to 200 mg/m^2^ and selected as the RP2D of ganetespib. After this phase I study, ganetespib 200 mg/m^2^ has been studied in several phase II studies as a single agent, and has shown to be well tolerated.

The most common toxicities were diarrhea and fatigue. Although there was no correlation with AUC or C_max_, diarrhea incidence appeared to increase with increasing doses of ganetespib, and it may serve as a PD biomarker for ganetespib. Diarrhea has also been observed with other Hsp90 inhibitors [18-21], suggesting that it may be a mechanism-based toxicity rather than an off-target effect. EGFR, a known client protein to Hsp90, is recognized to play a key role in intestinal epithelial integrity and restitution [22-24]. Consequently, proactive diarrhea management is incorporated in recent ganetespib clinical trials.

Two patients in the current study experienced treatment-related visual impairment, which were mild and transient. Hsp90 plays a key role in the folding of key signaling molecules required to maintain retinal function. Visual disorders, including blurred vision, flashes, delayed light/dark accommodation and photophobia, have been reported with other Hsp90 inhibitors, suggesting retinal injury [21,25-27]. It was recently postulated that high retinal exposure and the slow elimination rate of several Hsp90 inhibitors with hydrophilic properties led to induction of apoptosis in the retinal outer nuclear layer [28]. Over 400 patients have been treated to date with ganetespib in other studies. The incidence of treatment related visual changes is <3% (unpublished observation) suggesting that the physicochemical properties of ganetespib molecular structure may provide a favorable safety profile [12]. No formal ophthalmologic examination was required in this study.

Clinical activity of ganetespib was demonstrated in heavily pre-treated patients with metastatic cancers. Disease stabilization was generally associated with doses higher than 80 mg/m^2^. However, due to the limited response data, it was not possible to characterize the relationship between exposure to ganetespib and clinical activity. However clinical effect may be linked to the biological profile of the tumor since two patients, who presented with NSCLC and GIST and achieved SD, had tumors harboring BRAF G469A and *PDGFRA*^*D842V*^ exon 18 mutations, respectively. Interestingly, activated BRAF [29] and mutated PDGFRA [30] are known client proteins requiring Hsp90, and these oncogenes can be effectively degraded by Hsp90 inhibitors [30-32]. Ongoing clinical trials are currently focusing on identifying the predictors of response to ganetespib treatment, based on molecular characterization of tumor tissues.

The up-regulation of HSP70 is used as a marker of Hsp90 inhibition [21,33-36]. We have evaluated the levels of serum HSP70 as a surrogate of intracellular HSP70 induction [11]. Although ganetespib induced elevations in circulating HSP70, serum levels were variable and did not appear to correlate with the ganetespib dose. Thus, HSP70 up-regulation as a pharmacodynamic readout appears to be indicative of biological activity of the drug, but does not predict for tumor response. Similar observations have been reported in clinical trials of other Hsp90 inhibitors [18,37] that have typically investigated HSP70 up-regulation in PBMCs as part of their pharmacodynamic analyses. PBMCs were not evaluated in this study, since HSP70 expression in these cells had previously showed limited utility as a surrogate tissue for ganetespib activity in a separate trial (I. El-Hariry, unpublished data).

Ganetespib demonstrated linear PK with C_max_ and AUC increasing in proportion to dose. C_max_ and AUC were highly correlated indicating that C_max_ is a good predictor of overall exposure, presuming distribution and elimination processes are unaltered. Drug elimination is rapid relative to the dosing frequency. Overall variability in exposure is small to moderate, as represented by a coefficient of variation of 33.8% for clearance (the reciprocal of dose-normalized AUC).

## Conclusions

In conclusion, once weekly dosing of ganetespib is well tolerated. The RP2D is 200 mg/m^2^, and is associated with an acceptable safety profile. Based on these findings, multiple phase II studies have been initiated. Ganetespib is currently being investigated in a global randomized phase II/III study in combination with docetaxel in 2^nd^ line NSCLC patients.

## Abbreviations

DCR: Disease control rate; DLT: Dose limiting toxicity; GIST: Gastrointestinal stromal tumor; HSP70: Heat shock protein 70; Hsp90: Heat shock protein 90; MTD: Maximum tolerated dose; NSCLC: Non-small cell lung cancer; RP2D: Recommended phase II dose; SCLC: Small cell lung cancer.

## Competing interests

IE-H, FT, VMV, RB, MDK, YC, WG and TI are current employees of Synta Pharmaceuticals Corp. All other authors declare that they have no competing interests.

## Authors’ contributions

VMV and LSR conceived the study design. JWG, RNR, GAG and LSR contributed to patient recruitment and collection of data. JWG, RNR, GAG, IE-H, FT, VMV, RB, MK, YC, WG, TI and LSR analyzed and interpreted the data. JWG, IE-H and LSR prepared the manuscript. All authors read and approved the final manuscript.

## Pre-publication history

The pre-publication history for this paper can be accessed here:

http://www.biomedcentral.com/1471-2407/13/152/prepub
